# Context-Dependent Competition in a Model Gut Bacterial Community

**DOI:** 10.1371/journal.pone.0067210

**Published:** 2013-06-14

**Authors:** Eric J. de Muinck, Nils Chr. Stenseth, Daniel Sachse, Jan vander Roost, Kjersti S. Rønningen, Knut Rudi, Pål Trosvik

**Affiliations:** 1 Center for Ecological and Evolutionary Synthesis (CEES), Department of Biosciences, University of Oslo, Oslo, Norway; 2 Division of Epidemiology, Norwegian Institute of Public Health, Oslo, Norway; 3 NOFIMA The Norwegian Institute of Food, Fisheries and Aquaculture Research, Ås, Norway; 4 Institute of Clinical Medicine, University of Oslo, Oslo, Norway; 5 Department of Medical Biochemistry, Oslo University Hospital, Oslo, Norway; 6 Department of Paediatric Research, Oslo University Hospital, Oslo, Norway; 7 Department of Chemistry, Biotechnology and Food Science, University of Life Sciences, Ås, Norway; University of Illinois, United States of America

## Abstract

Understanding the ecological processes that generate complex community structures may provide insight into the establishment and maintenance of a normal microbial community in the human gastrointestinal tract, yet very little is known about how biotic interactions influence community dynamics in this system. Here, we use natural strains of *Escherichia coli* and a simplified model microbiota to demonstrate that the colonization process on the strain level can be context dependent, in the sense that the outcome of intra-specific competition may be determined by the composition of the background community. These results are consistent with previous models for competition between organisms where one competitor has adapted to low resource environments whereas the other is optimized for rapid reproduction when resources are abundant. The genomic profiles of *E. coli* strains representing these differing ecological strategies provide clues for deciphering the genetic underpinnings of niche adaptation within a single species. Our findings extend the role of ecological theory in understanding microbial systems and the conceptual toolbox for describing microbial community dynamics. There are few, if any, concrete examples of context-dependent competition on a single trophic level. However, this phenomenon can have potentially dramatic effects on which bacteria will successfully establish and persist in the gastrointestinal system, and the principle should be equally applicable to other microbial ecosystems.

## Introduction

*Escherichia coli* is a ubiquitous, albeit not abundant, member of the gastrointestinal (GI) microbiota [1]. Still, a healthy human will normally have more than one billion living in the intestine [2,3]. In addition to acute infections, *E. coli* colonization of the gut has been consistently linked to the chronic condition inflammatory bowel disease (IBD) [4], implicating particular types of strains in its aetiology [5–8]. It has also been proposed that competition between strains may be important due to the positive effects of the *E. coli* Nissle 1917 strain in IBD [9]. The wide spectrum of relationships between *E. coli* and humans highlights the importance of understanding colonization on the strain level and linking this to the dynamics of higher taxa.

In theory, and in contrast to a chemostat system, the fluctuating nutrient levels inherent in a batch culture can be used to promote coexistence of strains on a single nutrient [10,11]. These dynamics are a special case for pairs of species in which one (sometimes called a “gleaner”) has a higher growth rate at low nutrient concentrations and another (sometimes called an “exploiter” or “opportunist”) has a higher growth rate at high nutrient concentrations [12,13]. This relationship can be described in terms of the Monod equation μ= μ_max_s/(K_s_+s) [14] where the outcome of competition is determined by the maximum growth rate (μ_max_) and the Monod constant (K_s_), i.e. the nutrient concentration at which a species has a growth rate of μ_max_ /2. In the special case where one species has a higher μ_max_ while the other has a lower K_s_, there exists a nutrient concentration (s) at which the growth rates are equal. Below that concentration, the species with the lower K_s_ will win, while higher concentrations favor the species with the higher μ_max_. We will refer to this case of affairs as ‘species-pair with high u_max_ and low K_s_’ (sHULK). The exact value of s that in theory will permit coexistence is for practical reasons very difficult to achieve in a chemostat. In contrast, due to the inherent nutrient dynamics in the serial batch culture regime, fluctuations between high and low resource availability can produce conditions that favor high u_max_ species early on and the low K_s_ species at later stages as nutrients become depleted, thus promoting sustained coexistence. In fact, some experimental evidence does support this type of proposed interaction [15,16]. However, much controversy still remains concerning the conditions that lead to coexistence [17].

In the ecological literature, there is abundant evidence that interactions between pairs of species are dependent on the community context through indirect interactions with other species [18,19]. Such biotic interaction have been classified into two main types; density-mediated and trait-mediated indirect interactions (DMII and TMII respectively) [20]. The former describes the case where the density of one of a focal species pair is affected by a third party species, with effects cascading to the second of the pair. The latter pertains to a situation where a third species causes the interaction between a species pair to change due to phenotypic alterations. TMII has been documented in a number of experimental systems involving three trophic levels [21], as well as exploitative competitor pairs sharing either a common resource [22] or predator [23]. Although these studies focus on behavioral traits, the plastic phenotype also includes more subtle traits like changes in physiological state or gene expression.

Experimental work demonstrating DMII has been carried out using host-parasite systems [24] including exploitatively competing pairs of bacteria and their bacteriophages [25]. The host-parasite system is analogous to the predator–prey system, consisting of two distinct trophic levels. However, much less is known about context-dependent interactions among guilds of organisms on one trophic level. This is especially true when it comes to the GI microbiota, although probiotic bacteria have been thought to compete for nutritional substrates and thus alter the microbial structure of the gut [26].

Here, we present observations of *E. coli* strains isolated from a cohort of infants and a series of competition experiments with strains isolated over a two-year period from a single infant. Previous analysis of the fecal samples derived from this infant cohort discovered that *E. coli* colonization of the gut before the age of 1 year reduced the likelihood of IgE sensitization and that early colonization was likely to have originated from the mother [27–29]. Our ability to use unmodified strains that were isolated from a single infant for the competition experiments allows us to make some tentative connections to the intestinal ecosystem. By measuring life history traits [30] and conducting competition experiments using different serial transfer regimes (due to the tractability of this experimental protocol and because the intestinal system most likely experiences nutrient pulsing [31]), we demonstrate that colonization at the strain level may be explained by relatively simple models. However, our experiments also reveal a mechanism by which the *E. coli* colonization process may be dramatically changed through a dynamic nutrient environment and interaction with gut community members belonging to a different phylum. Specifically, we show that the relative abundance of members of Firmicutes and Bacteriodetes may have the potential to influence strain level competition in *E. coli*. The balance between Firmicutes and Bacteriodetes, has been linked to a number of diseases including type 1 and type 2 diabetes, IBD, and obesity [32–35]. Thus, insights into the colonization dynamics in the infant gut on high taxonomic levels [36] and the potential to influence patterns on lower taxa are of potentially great value [37]. Our results provide a specific illustration of the concept of context-dependent competition in bacteria on the same trophic level, where the outcome of competition between closely related strains during colonization can be determined by how the nutrient structure in the environment is modified by the established community.

Deciphering the complex association between genomic coding potential of a bacterial strain and its ecological phenotype is difficult, but some headway has been made in the case of *E. coli*, including some of the strains used in this study [38,39]. Here, we present genomic sequences of three strains representing the distinct ecological strategies we observed during phenotypic characterization and competition experiments. Comparing the genomes of these strains allowed us to generate new hypotheses of specific genetic factors that underpin these strategies.

## Results

### Growth rate and colonization

In general, infant GI-bacterial species appear to have shorter generation times when compared with adult GI-bacterial species [40]. This was attributed to faster growing organisms having an advantage during early colonization [41]. We measured the generation times of 23 different *E. coli* isolates under aerobic and anaerobic growth conditions (Table S1) and found that low generation times in aerobic culture have a tendency to coincide with early colonization (p=0.034, one-tailed Mann–Whitney U test) (Figure S1). That this effect was observed under aerobic but not anaerobic conditions (p=0.95) coincides with the aerobic environment thought to exist in the early infant gut [42]. Of note, no trade-off was found between anaerobic and aerobic growth rates for the collection of *E. coli* isolates. Instead, a significant positive correlation was found between maximal aerobic growth rate and maximal anaerobic growth rate (p<0.001) (Figure S2A). Further analysis revealed a strong relationship between an increase in anaerobic growth rate and difference between anaerobic and aerobic growth rates (p<0.001) (Figure S2C). One strain (EDM106) however, did not follow the strict relationship between anaerobic and aerobic growth rates and had a faster growth rate under anaerobic conditions than would have been expected (Figures S2A and S2B).

### Competition between strains isolated from an infant

In order to focus our investigation on strain competitiveness during colonization we looked more closely into the *E. coli* strain succession in the infant that harboured EDM106. We had isolated three distinct *E. coli* strains from this infant and it is likely that these isolates would have interacted with one another during the gut colonization process. A single strain was found at four days of age (EDM106), whereas EDM106 and EDM116 were found to coexist in the sample collected at one year of age (see materials and methods). At two years of age, both of these strains had been supplanted by EDM530. The three strains had no apparent clonal relationship, as determined from genome analysis (*below*), and they were the only *E. coli* strains found in the infant over the two year sampling period as determined from *mdh* fragment amplification and sequencing as described in [27]. The infant did not receive any antibiotic treatment during the sampling period.

Despite the abnormally high growth rate of EDM106 under anaerobic conditions, when compared with the entire collection of isolates, EDM106 had a longer anaerobic generation time (56.50±0.30 min.; mean ±s.e. with n=3) relative to EDM116 (50.97±0.32 min.; p < 0.01) and EDM530 (49.03±0.24 min.; p < 0.01, t-test). Thus, the final replacement event is in agreement with these measurements, assuming this is the main phenotype determining competition outcomes. Under aerobic conditions, EDM106 had a shorter generation time (38.12±0.35 min.) relative to both the EDM116 (40.98±0.49 min.; p < 0.01) and EDM530 (41.56±0.72 min.; p < 0.01) and thus, this strain fits the profile of an early colonizer with respect to aerobic growth rate [40,42].

Pair-wise competition experiments found that both EDM116 and EDM106 were individually outcompeted by EDM530 (Figure S3A and S4). If all three strains were competed together, EDM530 dominated the cultures (Figure S3B). Bacteriocins are a commonly ascribed determinant of bacterial intra-specific competition. Due to their potential effect and the fact that bacteriocin genes were discovered in the genomes, a susceptibility screen was performed. No effect on the strains used in the competition experiments was found. Plasmid transfer may also affect the competition experiments. The increased coverage depth of several contigs relative to the rest of the contigs of a genome sequence showed plasmid carriage by EDM106 and EDM116 (Table S2). In contrast, EDM530 does not seem to carry plasmids. After five days of co-culture of EDM530 and EDM106, no plasmid was isolated from EDM530, indicating a lack of plasmid transfer.

The co-occurrence of EDM106 and EDM116 in the sample taken at one year of age is difficult to explain given the substantially different generation times of these strains in anaerobic conditions. However, pair-wise competitions found that EDM106 and EDM116 were able to coexist in culture at stable densities for a substantial amount of time under both one and two day transfer regimes (Figure S5). The sHULK relationship described above could explain the observed competition outcomes between these strains in the batch culture regime. Growth rate measurements at different nutrient concentrations showed that these strains do in fact have this kind of relationship (Figure S6). Additionally, the co-culture showed an increased carrying capacity relative to the one expected from combining the single strain carrying capacities (p = 0.024, one-sample t-test) (Figure S7). Isoclines were generated as described in [43] using the measured carrying capacities and the relative abundances of strains in the co-culture, and this analysis supports that a stable equilibrium point can be reached by EDM106 and EDM116 (Figure S8).

EDM106 was outcompeted by EDM530 in serial batch culture. A series of competition experiments using different transfer times (12hours, 1day, 2days, 3days, and 4days) (Figure S4) found the same outcome under all conditions. There was, however, a positive non-linear relationship between increased time spent in low nutrient competition and number of transfers to out-competition (R^2^=0.95). This was further investigated using long term stationary phase cultures in which EDM106 eventually did outcompete EDM530 (Figure S9), supporting the hypothesis that EDM106 is a “gleaner” whereas EDM530 is an “exploiter” or “opportunist”.

### Competition between *E. coli* isolates in a model gut microbiota

**Figure 1:**
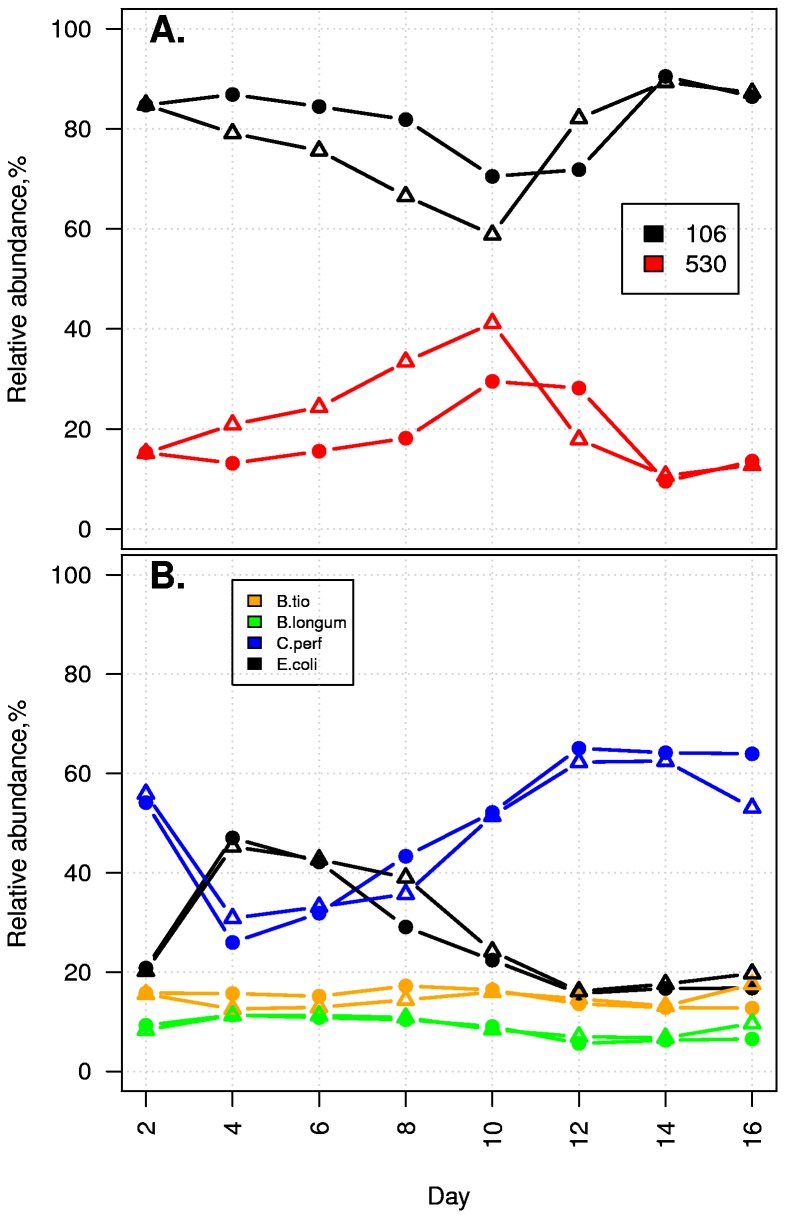
Intra-specific competition is modulated by the resident background community. Percent relative abundances are plotted as a function of time for the competitions between *C. perfringens* (C.perf), *B* *. thetaiotaomicron* (B.tio), *B* *. longum* and *E. coli* strains EDM106 and EDM530. Experiments were carried out in duplicate. A. Relative *E. coli* strain abundances. B. Relative species abundances. After day ten, competitive strain trajectories change, coinciding with *C. perfringens* dominance.

**Figure 2:**
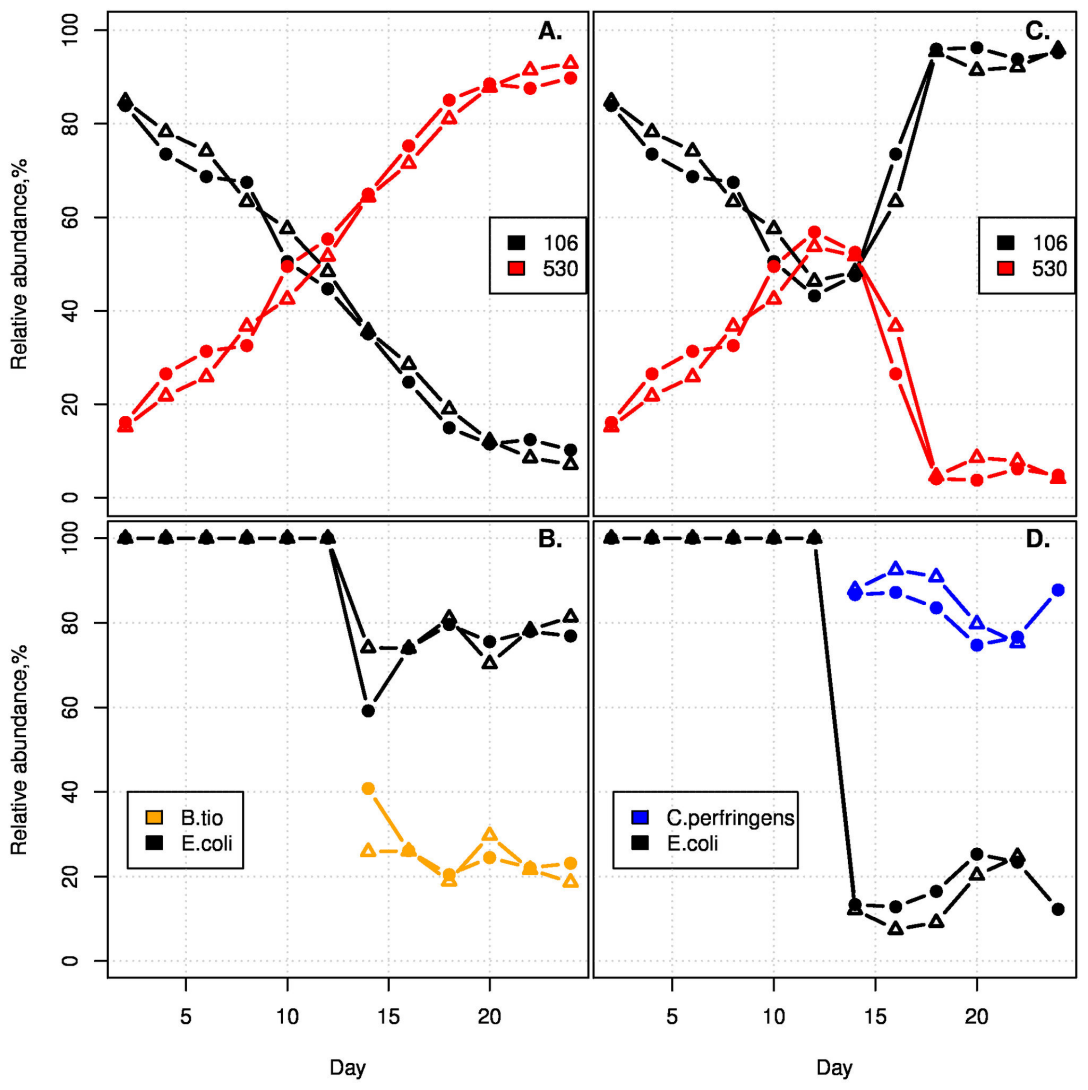
Intra-specific competition is context dependent. One competition was inoculated with *B* *. thetaiotaomicron* (B.tio) (A and B) while the other was inoculated with *C. perfringens* (C and D) at day 12 of the experiment. Experiments were carried out in duplicate. Top panels (A and C) show *E. coli* strain relative abundances. Bottom panels (B and D) show species relative abundances. Competition trajectories of strains EDM106 and EDM530 reversed in the competition inoculated with *C. perfringens* relative to when the strains were alone or inoculated with *B* *. thetaiotaomicron* .

In order to investigate whether the strain competitions would be influenced by the presence of a background community, we used a simplified model gut microbiota with species representing the four main phyla (Firmicutes, Bacteroidetes, Actinobacteria and Proteobacteria) inhabiting the human gut [44]. *Clostridium perfringens*, *Bacteroides* *thetaiotaomicron* , and *Bifidobacterium* *longum* were inoculated into the batch culture system along with either EDM106 and EDM530 or EDM116 and EDM530. Species and *E. coli* strain dynamics were then followed over time. During the initial stages of the competition, *E. coli* strain dynamics remained consistent with previously described observations and continued as such for the competition between EDM116 and EDM530 (Figure S10). However, EDM530 no longer continued on the trajectory towards dominance after day 10. Instead, EDM106 came to dominate (Figure 1). This result was replicated in an independent experiment (Figure S11). The change in *E. coli* strain competition dynamics seemed to occur in conjunction with *C. perfringens* dominance in the batch culture. In order to investigate if the structure of the background community would affect the *E. coli* strain competition we performed the exact same competition experiment but under microaerophilic conditions to depress the growth of anaerobic bacteria and favour *E. coli* dominance. Despite EDM106 having a higher aerobic growth rate than EDM530, EDM530 continued on the same competitive trajectory (Figure S12). This result gives further support to the notion that the configuration of the background community, and in particular the relative abundance of *C. perfringens*, is important for altering the *E. coli* strain competitive interactions. The effect of *C. perfringens* on strain competition was further investigated by reviving cryogenic stocks collected on day 10 of a competition between EDM106 and EDM530, and spiking these cultures with high doses (10: 1 volume ratio relative to *E. coli*, see File S4) of either *C. perfringens* or *B* *. thetaiotaomicron* (Figure 2). Previous work has demonstrated that *C. perfringens* is a very effective competitor in similar experimental conditions as those used in this study [44]. The competition trajectories remained unchanged in the competition inoculated with *B* *. thetaiotaomicron* (Figure 2A-B). However, the competition trajectories reversed in the competition inoculated with *C. perfringens* (Figure 2C-D) indicating that *C. perfringens* dominance is the causative factor of reversed trajectories*.*

### Nutritional and metabolomic profiling of competitions

**Figure 3:**
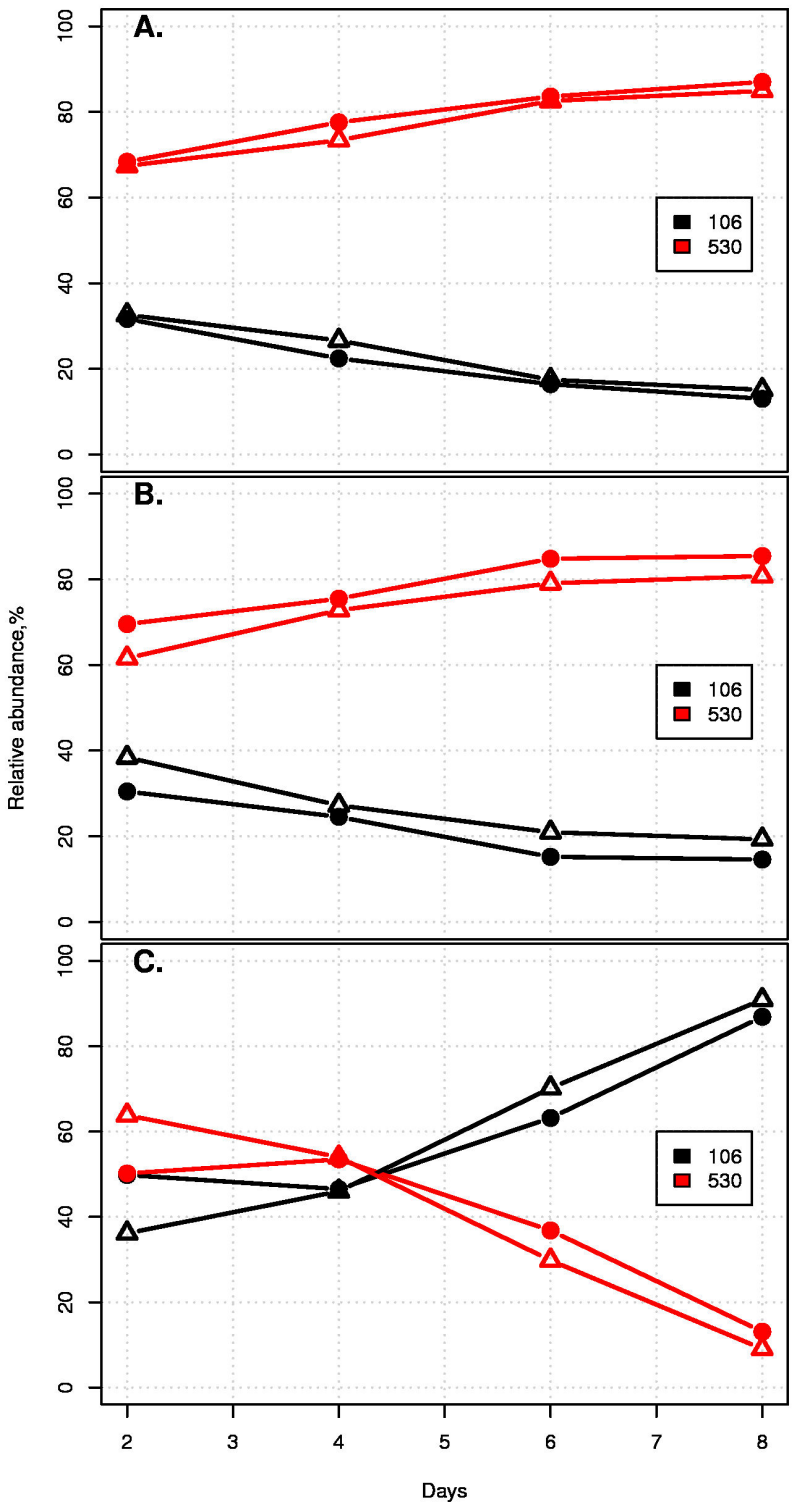
Strain competition outcomes are contingent on resource availability. After two days equilibration period in rich medium, aliquots were transferred into either 90/10 (A), 50/50 (B), 10/90 (C) rich/minimal salts medium (see Table S2 and File S4 for details) and the competitions were continued under these conditions. Experiments were carried out in duplicate. Strain competition trajectories shift in the low nutrient competition (C).

Investigation into potential reasons of altered strain competition outcomes by the addition of *C. perfringens* was begun by growing *C. perfringens* high cell density (12 hours) and then removing cells by filtration to create a spent rich medium that included any modulating factors released into the media. This procedure would not have depleted the medium of nutrients to such a degree that it would be expected to affect *E. coli* intra-specific competition (see below and Figure S13). Repeating the *E. coli* strain competitions in 90/10, 50/50, or 10/90 (spent/fresh) mediums did not produce any effects on competition outcomes (Figure S14) demonstrating that no modulating factors were being released by *C. perfringens*. The results from the long-term stationary phase culture experiment depicted in Figure S9, suggest that reduced resource levels can lead to a reversal of the competitive relationship between strains EDM106 and EDM530. Indeed, using rich/minimal-salts medium proportions of 90/10, 50/50, and 10/90 (Table S3) did find a reversal of the trajectories in the low nutrient (10/90) competition (Figure 3). HNMR spectra of the supernatants from these competitions identified differences linked to the competition outcomes, suggesting a chemical underpinning of the observations (Figure S15). HNMR signal peaks of complex samples can be attributed to many different types of molecules and therefore definitive identification is difficult.

**Figure 4:**
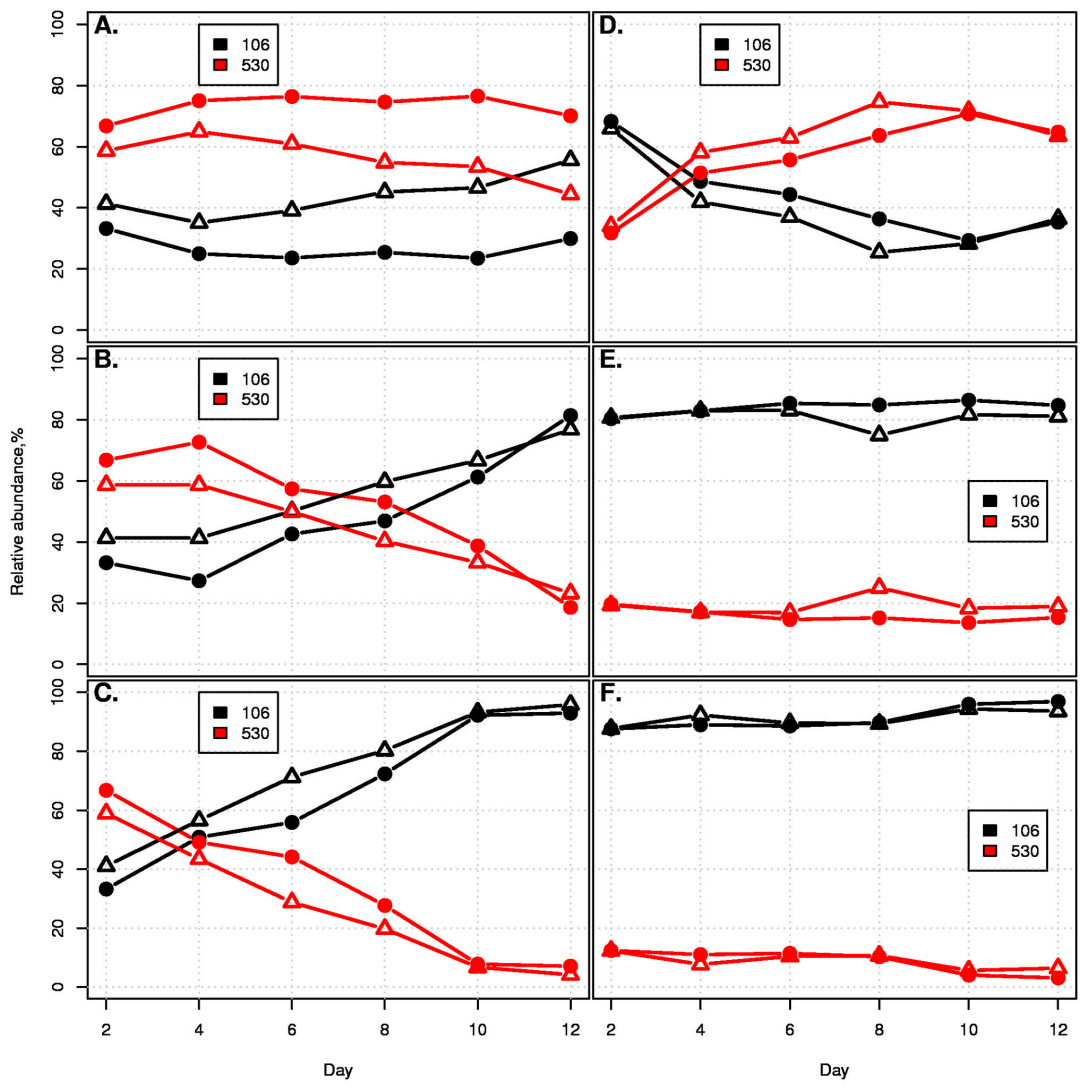
Peptones can act as a mediator of context-dependent competition. Two independent strain competition experiments were performed in duplicate in flasks containing minimal salts medium and different concentrations of peptone (Table S2). All experiments were carried out in duplicate. For the experiments shown in (A–C), The revived stocks were allowed two days equilibration period in Oxoid anaerobe basal broth, aliquots were then transferred into 90/10 (A), 50/50 (B), or 10/90 (C) peptone/minimal salts medium (A–C). Peptone concentration had a pronounced effect on the competition trajectories. For experiments shown in (D–F), a separate stock culture with a low relative starting abundance of strain EDM530 was inoculated into flasks into 200/10 (D), 50/50 (E), or 20/80 (F) peptone/minimal salts medium. Peptone concentration had a pronounced effect on the competition trajectories (p<0.001 for both experiments, logistic mixed effects model, see File S4).

We then performed competitions using high, intermediate, and low concentrations of glucose and peptone (Table S3), with the high nutrient regimes corresponding to the concentrations in the original rich medium to see if the reversal of competitive outcome was a result of strict density effects or related to specific compounds. EDM530 dominated under all three scenarios with glucose as the sole carbon source (Figure S16), indicating that the reversal was not strictly due to density effects. However, low concentrations of peptone resulted in a changes to the competition trajectories in two separate experiments (p < 0.001 for both experiments, logistic mixed effects model, see File S4) (Figure 4). To further assess the potential of peptone and glucose as mediators of the competition outcomes we measured the dynamics of these nutrients over a 48 hour period. Glucose levels were below the linear detection range of our assay by the first sampling point. However, the peptone measurements demonstrated that EDM530 was a more effective consumer of peptone at early time points when concentrations were high, whereas EDM106 was a more adept scavenger at later time points when concentrations were lower (Figure S13). *C. perfringens* behaved much like EDM530 with respect to peptone usage dynamics, with rapid depletion early on and incomplete usage at the end point of the experiment. These results suggest a mechanism by which the strain level competition may be modulated through ecological interaction with bacteria belonging to a completely different phylum. When *C. perfringens* rapidly lowers the amount of peptone available to the competing *E. coli* strains this will result in an environment that favors the ‘gleaner’ (EDM106) rather than the ‘exploiter’ (EDM530). Thus the competitive relationship between the *E. coli* is indirectly contingent on the biotic environment in which they find themselves by proxy of a nutritional intermediary, i.e. it is a context-dependent relationship.

### Genome comparisons

**Figure 5:**
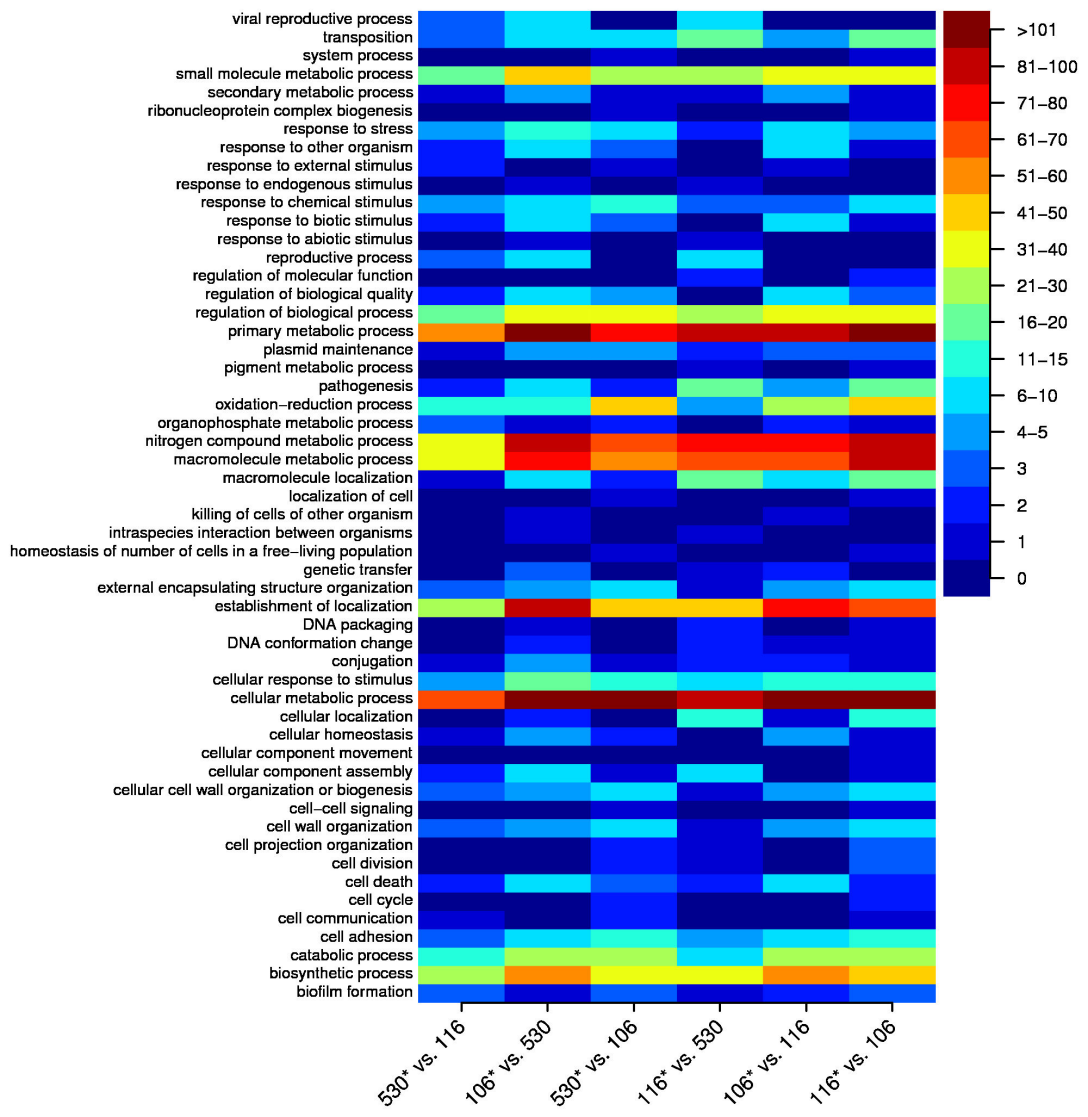
Functional categorization of differential gene content reveals similar biological enrichments using unique genetic elements. Columns represent the six possible pairwise comparisons of EDM530, EDM106, and EDM116. Asterisks next to the strain designations on the x-axis indicate the strain that is being represented by the column in each comparison. For each comparison, each genome was BLASTed against the other genome in the pair and a list was generated of genes that were unique to that strain. Each list was analysed using the Blast2GO software in order to assign the unique genes to biological process categories (ontology level 3). These categories are listed on the y-axis and the colours (see colour key, top right) represent the number of genetic elements in each biological process category.

Genomes were sequenced as described in materials and methods. A general comparison of the annotated genomes of the three sequenced strains found a core genome of 3535 genes (72% of the annotated total) that drops by 7.5% to 3271 genes if *E. coli* MG1655 (K12) is included in the comparison (Figures S17 and S18). An enrichment comparison of some candidate pathways identified potential functional categories (discussed below) that could influence competition results (Figure 5 and Files S1, S2 and S3). The large differences in gene content strongly indicate that these strains do not have a recent clonal relationship.

## Discussion

It would most likely be a question of “who gets there first” that determines the colonization pattern in the intestine. In most cases, it would be the mother who has first opportunity of exposing the fetus to the new microbiota. Still, growth rate has been previously linked to intestinal colonization [40], and we found a tendency of early colonizers to have a faster aerobic growth rate than late colonizers (Figure S1). Early colonizers of the infant gut are often a combination of aerobic and facultative anaerobic bacteria that use up the available oxygen and thus allow for the succession to obligate anaerobes which dominate the gut flora in the mature intestine [42]. The positive correlation between the aerobic and anaerobic generation times suggest that some strains are intrinsically more efficient at using nutrients than other strains regardless of whether they are in an aerobic or anaerobic environment (Figure S2A). The relationship discovered between the increase in minimal generation times and the difference between the anaerobic generation times and aerobic generation times (Figure S2C) suggests that metabolic efficiency reaches a plateau as a strain’s ‘intrinsic’ doubling time decreases. This could signal a physical constraint on efficient nutrient utilization that is independent of internal cellular machinery.

### Isolate competitions

The maximal growth rates of the three strains used in competitions matched the previously described pattern of colonization with the faster growing strain under aerobic conditions isolated from an earlier sample. Despite different growth rates of two of the isolates (EDM106 and EDM116) stable co-existence was observed in both one and two day transfer regimes. These observations can be explained by two different models. The first model invokes the sHULK relationship which would allow the two strains to be competitively superior in different phases of the batch culture. This would be in accordance with our growth rate measurements (Figure S6), and is a previously reported mechanism for maintenance of coexistence [10]. The second model is based on the observed increase in carrying capacity of the co-culture. If between-strain competition is weaker than within-strain competition there is a theoretical equilibrium point for coexistence (Figure S8). This state can be brought about cooperative interactions like cross-feeding, which is indicated in by the increased productivity in the co-culture (Figure S7). Acetate cross-feeding has been reported to evolve readily in *E. coli* cultures [45], but from our data we cannot determine the exact nature of the interaction between EDM106 and EDM116. Neither model excludes the other, but further experimentation is required for uncovering the mechanism of coexistence. However, the fact that these strains were found to coexist in the infant gut, as well as in co-culture suggests that they could occupy overlapping niches in their natural environment, and that the observed interaction is ecologically relevant.

In contrast, competitions with EDM106 and EDM530 under several different transfer regimes were consistently dominated by EDM530 (Figure S4). We did find a strong positive correlation between transfer rate and the number of transfers to outcompetition, and long term stationary cultures were dominated by EDM106 (Figure S9). The results presented in Figure 3 suggest that the eventual competitive outcome in the long term stationary cultures was not due to cell death, but rather that it was caused by an altered competitive interaction brought on by a changed nutrient environment. These results fit a model where the exploiter gains ground during the log period of growth while the gleaner does so during stationary phase. This hypothesis is supported by peptone usage measurements that further categorize EDM106 as a gleaner that can better use lower concentrations of peptone while EDM530 is an exploiter that is more optimized for faster utilization of higher concentrations of peptone (Figure S13). When all the competition results are taken together, we were able to replicate the outcomes of strain competition observed in the actual infant gut but this does not mean that these were the actual factors responsible for the outcomes in that complex environment. An additional consideration when evaluating these results is the detection threshold inherent in our quantification methodology. Using traditional serial dilution techniques, it is only possible to reliably determine very large abundance differences between populations of bacteria (i.e., log-fold). Our methodology (as presented in [46]) accurately measures fine scale relative abundances. However, this method does not allow us to conclude that a species or strain has been excluded from the culture because there is a detection limit around 5% relative abundance.

### Context-dependent competitive effects

Competition between EDM106 and EDM530 in the model gut background found that the competitive trajectories of the strains changed as *C. perfringens* became dominant (Figure 1). This indicates that the community structure of the surrounding species can modify the interaction between competing strains. While this may seem intuitive, examples of these types of interactions in bacterial systems are, to our knowledge, lacking.

The altered strain interactions in the presence of *C. perfringens* (and not *B* *. thetaiotaomicron* ) suggested that the *C. perfringens* was modifying the environment through either resource consumption or by releasing a factor to which EDM530 was more susceptible (Figure 2). The latter hypothesis was rejected after repeating the *E. coli* strain competitions in the presence of filtered supernatant failed to reverse the competition trajectories (Figure S14). HNMR spectroscopy also failed to identify any specific compound affecting the competitions. However, simply lowering the starting nutrient concentration resulted in the same change in competitive dynamics (Figure 3) as observed when *C. perfringens* was added to the medium. We were able to attribute this effect to peptone availability (Figure 4 Figure S13). These observations are in accordance with growth rate measurements that suggest a sHULK relationship between EDM106 and EDM530. They also suggest a concrete mechanism through which context-dependent interactions can occur between organisms on one trophic level.

Dogma dictates that organisms evolve towards maximal metabolic efficiency, but this does not explain how a metabolically effective organism can be at a disadvantage when a resource is abundant. However, a phenotype has been observed in several species of bacteria, including *E. coli*, showing growth inhibition in the presence of high concentrations of certain amino acids or combinations thereof [47]. It may also be the case that different strains have different abilities when it comes to maintaining an optimal intracellular nitrogen-carbon balance while growing on nitrogen rich compounds. These hypotheses may provide insight into reasons why high nutrient concentrations could affect the observed strain level competitive dynamics. *C. perfringens* is known to be able to drastically alter environments rich in amino acids because of enhanced proteolytic capabilities and high growth rates [48,49]. This may, in turn, change the intra-specific competitive interaction in favour of the low K_s_ strain rather than the high µ_max_ strain.

It is difficult to classify the context-dependent interaction that we observed as either TMII or DMII. While the density effect of *C. perfringens* on the abundance of the two *E. coli* strains in co-culture is evident, the effect on the strain interaction does not seem to be density dependent (Figure S16). Rather the effect is mediated through an environmental intermediary. It stands to reason that this would alter the physiological state, and hence the phenotype, of the *E. coli* strains, possibly in a differential manner, but that is not something we can conclude based on our data. Even so it would be incorrect to call this strain competition strict TMII since there is no direct biotic link between the context-dependent effect on *E. coli* and the intervening species. The literature provides several cases similar to the one described here, where a third species alters the interaction between two others indirectly through the environmental context, but this phenomenon has mostly been classified as TMII [19]. TMII, however, involves a very different mechanism where species A causes a phenotypic change in species B, thus altering the interaction between species B and C. Furthermore, the systems that have been studied for this purpose generally contain at least two species that are directly linked by a consumer-resource relationship. This is not the case in many bacterial systems where resource competition is presumed to be the predominant form of biotic interaction [50]. In these kinds of systems the environmental context is likely to be an important mediator of context-dependent effects. Thus, we suggest a third category of context-dependent higher order interaction, EMII (environmentally mediated indirect interaction), to describe this set of cases.

### Genome comparison

Previous investigation into colonization determinants of *E. coli* strains has mostly focused on adhesins and other factors that mediate host interactions. This is understandable considering the importance of pathogenic *E. coli* in human health. However, gut bacteria reside in complex communities in which bacteria-bacteria interactions occur mainly through resource competition, and the term virulence deserves, and has begun to receive, a broader definition that includes metabolic capabilities [51,52]. This is especially important in the case of *E. coli* because commensal and pathogenic strains share so many genomic features [53].

A general genome comparison of the three strains competed in this study found the number of genes in core genome, 72% of the annotated total, were well within the normal variance seen between *E. coli* genomes [54]. Investigation into several gene pathways hypothesized to be important for competition in our model system found differences between strains in peptide uptake and utilization, sugar uptake and utilization, and quorum sensing pathways (Files S1, S2 and S3). EDM106 is enriched compared with EDM530 for small molecule and secondary metabolic processes (Figure 5). This strain also encodes a unique putative oligo-peptide ABC transporter that could help explain the increased peptide affinity at low concentrations. Competition outcomes could also be influenced by strain specific differences in quorum sensing [55]. EDM106 has the *luxS* gene for production of autoinducer-2, but lacks a functional suite of *lsr* genes for quorum response. ‘Signal blind’ mutants had been found to have higher fitness than their wild type parent strain when present as a minority [56,57]. The relative enrichment of stress response genes of EDM106 (Figure 5) may help explain the advantage of EDM106 later in the competition, when toxic metabolic by-product concentrations are high, although further work would be required to make this claim.

EDM530 is enriched for oxidation reduction processes relative to both EDM106 and EDM116 which probably relates to the high anaerobic growth rate relative to the other two strains (Figure 5). EDM530 also encodes several unique systems for transmembrane transport and catabolism of sugars, including the *yihO* gene encoding a glucuronide transporter. This transporter could have impacts on competition and colonization in the infant gut since glucuronide is a major carbon source for *E. coli* in the intestine [58,59].

Interestingly, the enrichment analysis (Figure 5) found that the three strains in this study are unique in many respects, yet retain a gene content profile at the higher biological process level that is consistent. The ecological strategies represented by our ‘gleaner’ (EDM106) and ‘exploiter’ (EDM530) entail quite different lifestyles. E.g. the two strains may be able to use the same resources, but an ecological ‘exploiter’ would typically use low specificity and low affinity transporters representing an adaptation towards “feast” conditions, but resulting in reduced competitiveness during nutrient starvation [60]. Relatively slower growers, represented by the ecological ‘gleaner’, tend to have high affinity transporters, making them competitive in low nutrient environments, while at the same time making them susceptible to saturation or toxic effects when resources are plentiful [61]. The fact that these strains employ different genetic elements to achieve similar biological functions could represent underlying adaptation to the ecological niche, and is in agreement with an sHULK interpretation of the strain-pair relationship.

### Strengths, limitations and concluding remarks

The inherent limitations of our model system make it all the more surprising that we found some of the same competitive outcomes that were observed in the gut environment. Several pathways were identified in the genomes that could have influenced competitive outcomes and could lead to further investigations to solidify a bridge between gene content and competitive outcomes in the natural environment. It is difficult to ascribe our results to particular genes or pathways, especially due to the number of unique genes that are annotated as hypothetical proteins in each of the strains. However, we do provide genomic profiles of an ecological gleaner in comparison with two exploiter phenotypes. Since the genomic sequences provided here are linked with extensive ecological metadata, this information could form a valuable basis for comparison in studies of related phenomena. We also present evidence of context-dependent competition in bacteria, and we propose mechanisms that can promote this phenomenon. Understanding the population ecology of gut bacteria is of increasing importance with increased use of antibiotics and probiotics for therapeutic ends without knowledge of possible cascading effects [62].

Our observations offer a general mechanism by which the fine scale dynamics of microbial communities can be determined by biotic processes. In a system where a high degree of exploitation competition takes place on several taxonomic levels, the ability of keystone taxa to remodel the abiotic environment may have profound effects on community structure. In the present case we can easily envision two scenarios where one of the two *E. coli* strains outcompetes the other, depending who is dominating the background community (*Clostridia* or *Bacteroides* ). Context-dependent competition most likely represents a general phenomenon where community composition at high taxonomic levels determines the outcomes of strain level colonization processes by remodelling the environment to become more permissive to some strains than others. This suggests a mechanism by which temporal changes in a limiting nutrient concentration can promote coexistence by changing the competitive interactions between strains. Given two species with an sHULK relationship competing in an environment where nutrient concentrations fluctuate, periods of high resource availability would favor one member of the pair, whereas low nutrient conditions would favor the other. Context-dependent competition may be especially important in the GI-system since it is subject to a pulse-like nutrient regime where key resources alternate between high and low concentrations.

## Materials and Methods

### Strains, life history trait measurement and competitions

Strains were obtained from the IMPACT cohort and all are described as part of that study except EDM530 [27]. EDM530 was isolated after the previous study. The phylogenetic relationship between the strains, based on whole genome sequences, has been described elsewhere [38]. The model background microbiota has been described elsewhere [44]. Growth rates and competition experiments (see File S4 for details) were performed in Oxoid anaerobe basal broth, a medium designed to accommodate GI bacteria, unless otherwise stated. Growth rates were measured in triplicate by monitoring OD600 with a Bioscreen (Oy Growth Curves Ab Ltd, Finland). Carrying capacities were measured by freeze drying and weighing (see File S4 for details).

### Determination of co-existence of EDM106 and EDM116 in one year fecal sample

A 600 bp region of the *mdh* gene was sequenced directly from the DNA extracted from the fecal sample. From the electropherogram we identified mixed peaks in 7 positions that indicated two co-existing strains with one in lower abundance. Visual decomposition of the mixed electropherogram into two sequences and subsequent alignment with the pure sequences of EDM116 and EDM106 found perfect identity for both subsequences. Multiple linear regression of the mixed spectrum against the strain unit spectra (see below) found relative abundances of 84% and 16% for EDM116 and EDM106 respectively (R-squared=0.975, p<0.0001), providing evidence of the co-existence of these two strains in the one year sample.

### Sample processing and quantification of relative abundances

DNA extraction, PCR amplification, and sequencing of *mdh* and 16s rRNA gene fragments were performed using previously described methods [27,46]. The relative abundances of strains and species in the competitions were determined by multivariate decomposition of mixed *mdh* or 16SrRNA gene sequence electropherograms as previously described [27,46].

### Bacteriocin screening and plasmid detection

All strains were subjected to a bacteriocin screen using the overlay method with each used both as an overlay and stab inoculate. Plasmid extraction from the three strains used in the competitions was carried out using the Promega (Madison, WI, USA) Wizard® *Plus*SV Miniprep system and isolates were visualized on a gel.

### Proton nuclear magnetic resonance (HNMR) spectra

Samples were spun down at 12,000 rpm (13,400g) at 4^°^C and the supernatant was removed and combined with potassium salts buffer at pH 7.4 and TSP as an internal standard. NMR spectra were obtained from a Bruker Avance 600 NMR spectrometer. 1d NOESY spectra were acquired with presaturation for water suppression. The spectra were referenced to the TSP signal at 0 ppm, baseline corrected and normalized to a constant sum of 1. Prior to PCA, the spectra were mean centered and scaled to unit variance. All statistical computations were carried out using R (R Development Core Team, 2011).

### Genome sequencing and assembly

The genomes of *E. coli strains* EDM530, EDM106, and EDM116 were sequenced using Roche 454 GS (FLX Titanium) pyrosequencing according to standard protocol. Number of contigs, median depth and N50 were 198, 17.5 and 1209, 585.8, 17.5 and 4007, and 864, 8.2 and 2714 for the strains, respectively. De Novo assembly was performed using Newbler v 2.3 with default settings and contigs were annotated with RAST [63]. Characterization of shared and unique gene content was done by BLASTing all annotated genes of all strains against one another using 85% sequence identity and an e-value cutoff of <1e^-25^ for assignment of presence (see Files S4 and [38] for details). Gene enrichment analysis and gene ontology (GO) assignment were carried out using the Blast2GO software package using the SEED categorization derived from ontology level 3 biological process assignments [64]. All DNA sequences have been submitted to the European Nucleotide Archive database with sample accession numbers ERS155058 (EDM106), ERS155052 (EDM116), and ERS155050 (EDM530).

## Supporting Information

Figure S1 Early colonizers have a tendency towards faster growth rates than later colonizers under aerobic conditions. 23 different *E. coli* strains were categorized as either early or late colonizers (see Table S1 for categorization) for comparison of growth rates in aerobic and anaerobic conditions. There was a tendency for early colonizers to have a shorter generation time than late colonizers in the aerobic environment (p = 0.03, one-tailed Mann–Whitney U test).

Figure S2 Positive correlation between aerobic and anaerobic growth rates. (**A**) A strong positive correlation is seen between aerobic and anaerobic growth rates. However, strain EDM106 (red) does not seem to follow the trend of the other strains. (**B**) This strain (red) deviates from the trend by more than two standard deviations and is due to a faster aerobic growth rate than should be for its anaerobic rate. (**C**) Even with this deviation, strain EDM106 and all the others show a strong relationship between anaerobic generation time and the difference between anaerobic and aerobic generation time. This indicates that fast growing strains are optimized for both conditions.

Figure S3 Strain EDM530 dominates in competition with strain EDM116 as well as with the combination of strains EDM106 and EDM116. (**A**) Strain EDM116 vs. EDM530. (**B**) Strain EDM106 vs. EDM116 vs. 530. Batch culture competitions were performed in Oxoid anaerobe basal broth. See File S4 for details. The two independent replicates are represented by solid circles and open triangles.

Figure S4 Strain EDM106 was dominated by strain EDM530 under four different transfer regimes. Cultures were transferred every 12 hours (**A**), 24 hours (**B**), 2 days (**C**) or 3 days (**D**). Batch culture competitions were performed in Oxoid anaerobe basal broth. The two independent replicates are represented by solid circles and open triangles.

Figure S5 Strains EDM106 and EDM116 coexist in one day and two day culture regimes. Batch culture competitions of strains EDM106 and EDM116 were performed in Oxoid anaerobe basal broth (rich medium). (**A**) One day transfer regime. (**B**) Two day transfer regime. The two independent replicates are represented by solid circles and open triangles.

Figure S6 Strain EDM106 has shorter generation times under low nutrient conditions than strain EDM116 and strain EDM530. Doubling times were measured for each of the three strains with different concentrations of Oxoid anaerobic basal broth medium (% medium) and minimal salts solution under anaerobic conditions. Bars are ±1 s.e. The fitted lines are local polynomial regressions.

Figure S7 Dry weight of single strain and mixed cultures. Carrying capacity of competitor strains in dry weight (grams) per 30ml medium (± s.e). The co-culture has a higher carrying capacity (p = 0.024, one sample t-test) than expected from combining the mean carrying capacities of the individual strains in the approximate proportions provided by the competition experiments (55% strain EDM106 and 45% strain EDM116 during co-culture).

Figure S8 Isocline plot based on carrying capacities of strains EDM106 and EDM116. Zero isoclines of carrying capacities were calculated using the approximate proportions provided by competition experiments. The y-axis (N_1_) is the population density of EDM106 in grams/30ml, while the x-axis (N_2_) is the density of EDM116. K_1_ and K_2_ are the carrying capacities of EDM106 (19.9 g/30ml) and EDM116 (21.6 g/30ml), respectively. The carrying capacity used for the co-culture was 21.8 g/30ml. The alphas signify the competition coefficients, i.e. the effect of EDM116 on EDM106 (α_12_) and vice versa (α_21_). The intersecting isoclines show a stable equilibrium point to which the two populations are attracted (arrows) and where they can co-exist.

Figure S9 Long term stationary phase cultures of strains EDM 106 and EDM530. Cultures were sampled for relative abundance measurement every two days but no fresh media was added to the cultures. The two independent replicates are represented by solid circles and open triangles.

Figure S10 The presence of a background flora does not change the outcome of competition between strains EDM116 and EDM530. Competition between *C. perfringens* (C.perf), *B* *. thetaiotaomicron* (B.tio), *B* *. longum* and *E. coli* strains EDM116 and EDM530. Each experiment was performed in duplicate. (**A**) *E. coli* strain competition trajectories. (**B**) Relative species abundances at corresponding time-points. Batch culture competitions were performed in Oxoid anaerobe basal broth. The two independent replicates are represented by solid circles and open triangles.

Figure S11 Replicate of experiment presented in Figure 1
: Intra-specific competition is modulated by the resident background community. Percent relative abundances are plotted as a function of time for the competitions between *C. perfringens* (C.perf), *B* *. thetaiotaomicron* (B.tio), *B* *. longum* and *E. coli* strains EDM106 and EDM530. Experiments were carried out in duplicate. (**A**) Relative *E. coli* strain abundances. (**B**) Relative species abundances. After day ten, competitive strain trajectories change, coinciding with *C. perfringens* dominance. The two independent replicates are represented by solid circles and open triangles.

Figure S12 Micro-aerophilic conditions increase *E. coli* predominance in the model microbiota, resulting in unmodulated *E. coli* strain competition trajectories. Percent relative abundances are plotted as a function of time for the competitions between *C. perfringens* (C.perf), *B* *. thetaiotaomicron* (B.tio), *B* *. longum* and *E. coli* strains EDM106 and EDM530. Experiments were carried out in duplicate. (**A**) Relative *E. coli* strain abundances. (**B**) Relative species abundances. After day ten, competitive strain trajectories do not change. This result occurs despite the higher aerobic growth rate of strain EDM106 relative to EDM530. The two independent replicates are represented by solid circles and open triangles.

Figure S13 Peptone usage by strains supports categorization of EDM106 as a ‘gleaner’ and EDM530 as an ‘exploiter’ phenotype. Overnight cultures were inoculated into fresh medium and samples were taken at 6, 12, 24 and 48 hours. Absorbance at 230nm was measured over time to determine peptone concentrations (see File S4). In the EDM106 culture peptone levels were consistently higher throughout the first 24 hours compared with cultures of EDM530, EDM530/EDM106 co-culture, and *C. perfringens* (asterisk indicates p<0.05). At 48hours, peptone levels were lower in the cultures containing EDM106 than cultures without EDM106 (circled asterisk indicates p<0.05). Glucose concentrations were also measured but were below the linear detection range of the assay for all strains by 6hours.

Figure S14 Spent medium from a *C. perfringens* culture did not affect competition between strains EDM106 and EDM530. Investigation into potential factors released into the media was performed by growing *C. perfringens* to saturation (14 hours) in Oxoid anaerobic basal broth and then removing cells by filtration to create a spent rich medium. (**A**) 90/10, (**B**) 50/50, or (**C**) 10/90 (spent/fresh) medium proportions were then used for the *E. coli* strain competitions. The two independent replicates are represented by solid circles and open triangles.

Figure S15 Competitions in low nutrient media produce qualitatively different media than more concentrated media. PCA of normalized HNMR spectra of competition time points in 90/10 (black dots), 50/50 (red dots), 10/90 (green dots) minimal salts (W)/ Oxoid anaerobic basal broth (rich medium). Clustering differentiates the spectra of the 10/90 and 50/50 (W)/rich medium competition supernatants compared with 90/10 medium.

Figure S16 Strain EDM106 and EDM530 competition trajectories remain unchanged at different levels of glucose availability. Minimal salts medium with different amounts of glucose as the sole carbon source was used for the competitions (Table S2). Revived frozen stocks from day 10 of strain EDM106 and strain EDM530 competition were used to start the competition. After two days equilibration in Oxoid anaerobic basal broth (rich medium), aliquots were transferred into either (**A**) 90/10, (**B**) 50/50 or (**C**) 10/90 glucose/minimal salts medium, relative to the amount of glucose in the rich medium (Table S2). The two independent replicates are represented by solid circles and open triangles.

Figure S17 Comparison of gene content between strains. The three strains had totals of 4,192 genes (S106), 4,237 genes (S116) and 4113 genes (S530). The relative percents of unique genes found S106 with the largest (9.7%), 6.6% for S116, and S530 had the least (4.7%). Core genes represented 72% of the annotated total. See Supporting Data Files S1, S2 and S3 for listings of non-core gene annotations.

Figure S18 Comparison of gene content between strains. The three strains had totals of 4,192 genes (S106), 4,237 genes (S116) and 4113 genes (S530). The relative percents of unique genes found S106 with the largest (9.7%), 6.6% for S116, and S530 had the least (4.7%). Core genes represented 72% of the annotated total.

Table S1 Table of isolates examined in this study with growth rates and standard errors. Colonization categorization is also listed in the final column. Strains isolated before the first two weeks of are categorized as early colonizer strains and strains isolated after two weeks of age are categorized as late colonizers. The isolates marked with an asterisk in the Category column have a sister isolate from the same sample. Means of the two independent growth rate measurements were used for testing.

Table S2 Listing of high depth contigs from the genome **sequencing**.

Table S3 Table of media preparations used for the competitions. Formula show the complete recipe Oxoid anaerobic basal broth and the columns show the recipe for 200%, 90%, 50%, 20% and 10% concentrations. The competitions with glucose or peptone as sole carbon sources were used proportions relative to the complete Oxoid anaerobic basal broth. Peptone broths A-C used Bacto™ Peptone from BD Biosciences. Peptone broths D–F used Fluka peptone from Sigma-Aldrich.

File S1 RAST annotation of genes found in strain EDM106 and not in strains EDM116 or EDM530.

File S2 RAST annotation of genes found in strain EDM116 and not in strains EDM106 or EDM530.

File S3 RAST annotation of genes found in strain EDM530 and not in strains EDM106 or EDM116.

File S4 Supplementary methods file with detailed description of experimental procedures.
