# Supplementary figures and images for: Context-Dependent Competition in a Model Gut Bacterial Community
Source: PLoS One. 2013 Jun 14;8(6):e67210. doi: 10.1371/journal.pone.0067210 (PMC3683063; doi:10.1371/journal.pone.0067210)

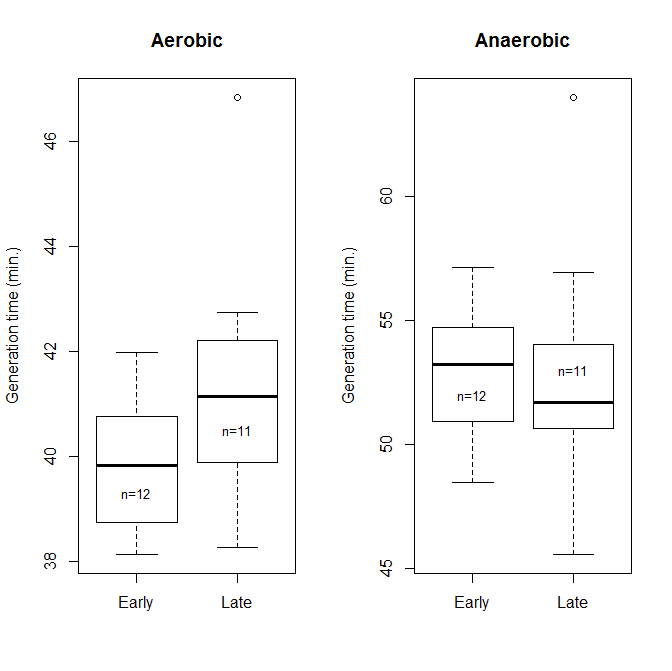

Supplement: Figure S1 — 23 different E. coli strains were categorized as either early or late colonizers (see Table S1 for categorization) for comparison of growth rates in aerobic and anaerobic conditions. There was a tendency for early colonizers to have a shorter generation time than late colonizers in the aerobic environment (p = 0.03, one-tailed Mann–Whitney U test). [file pone.0067210.s001.tiff]

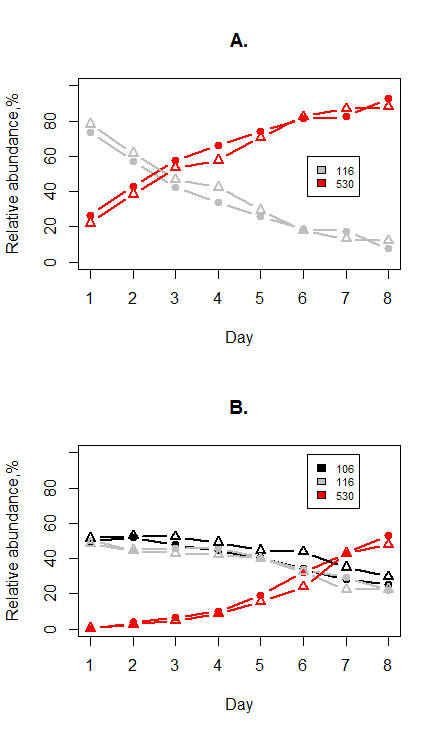

Supplement: Figure S3 — (A) Strain EDM116 vs. EDM530. (B) Strain EDM106 vs. EDM116 vs. 530. Batch culture competitions were performed in Oxoid anaerobe basal broth. See File S4 for details. The two independent replicates are represented by solid circles and open triangles. [file pone.0067210.s003.tiff]

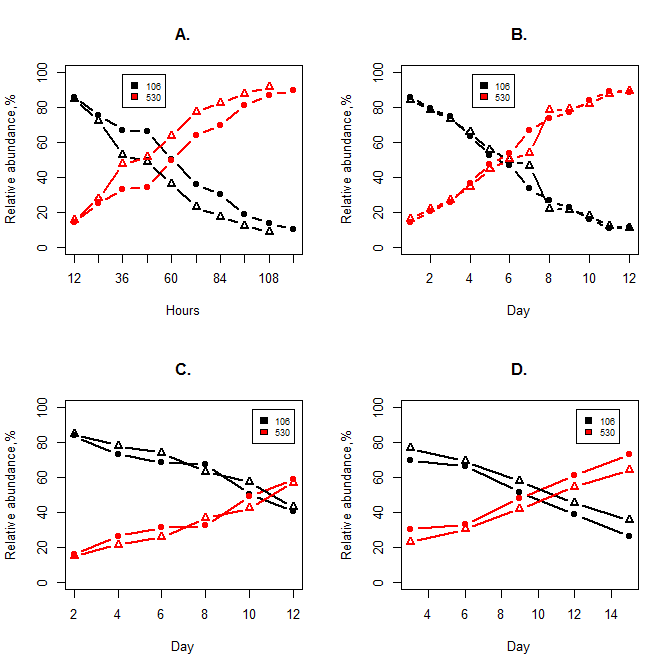

Supplement: Figure S4 — Cultures were transferred every 12 hours (A), 24 hours (B), 2 days (C) or 3 days (D). Batch culture competitions were performed in Oxoid anaerobe basal broth. The two independent replicates are represented by solid circles and open triangles. [file pone.0067210.s004.tiff]

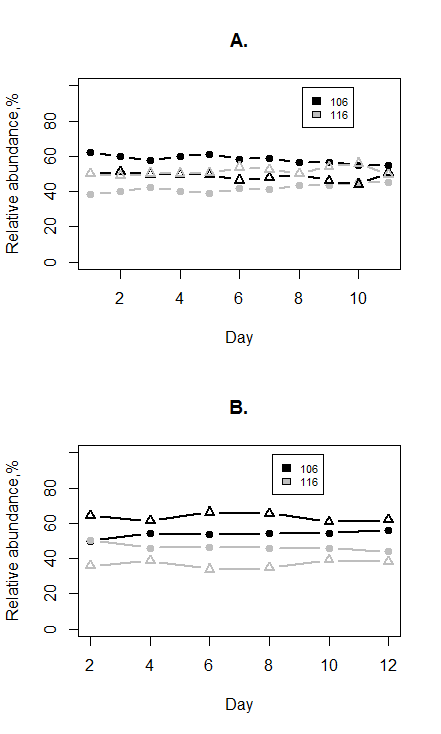

Supplement: Figure S5 — Batch culture competitions of strains EDM106 and EDM116 were performed in Oxoid anaerobe basal broth (rich medium). (A) One day transfer regime. (B) Two day transfer regime. The two independent replicates are represented by solid circles and open triangles. [file pone.0067210.s005.tiff]

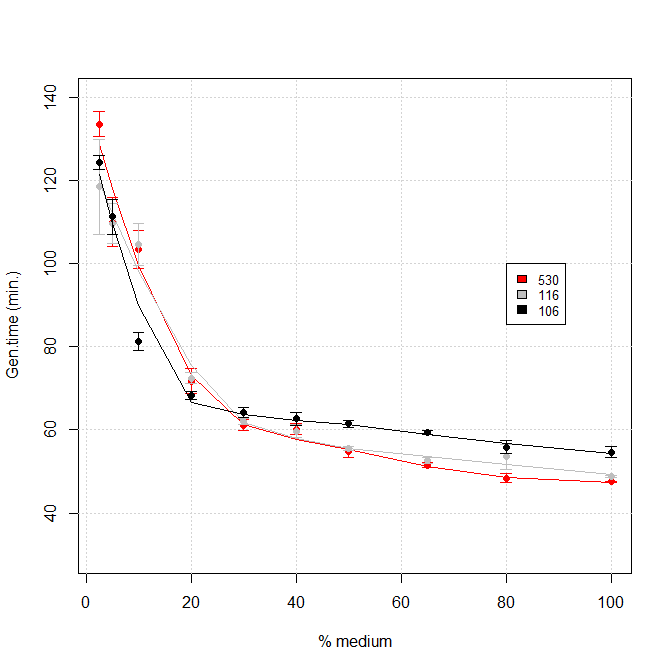

Supplement: Figure S6 — Doubling times were measured for each of the three strains with different concentrations of Oxoid anaerobic basal broth medium (% medium) and minimal salts solution under anaerobic conditions. Bars are ±1 s.e. The fitted lines are local polynomial regressions. [file pone.0067210.s006.tiff]

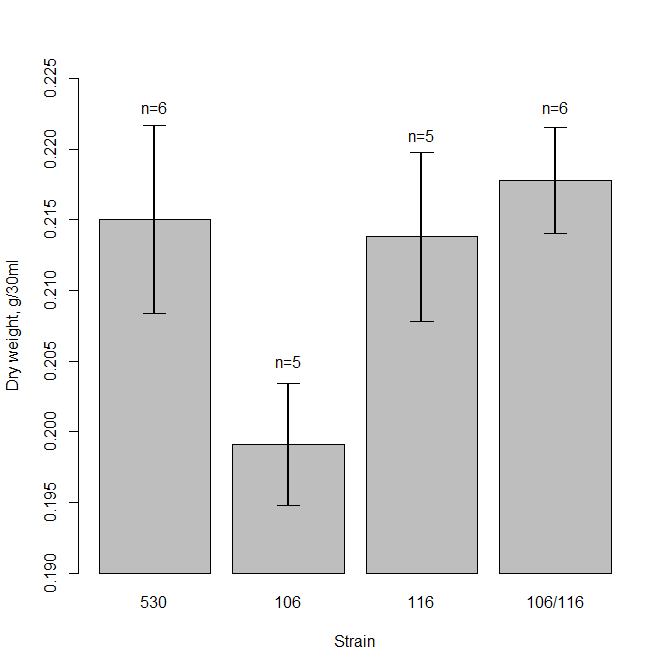

Supplement: Figure S7 — Carrying capacity of competitor strains in dry weight (grams) per 30ml medium (± s.e). The co-culture has a higher carrying capacity (p = 0.024, one sample t-test) than expected from combining the mean carrying capacities of the individual strains in the approximate proportions provided by the competition experiments (55% strain EDM106 and 45% strain EDM116 during co-culture). [file pone.0067210.s007.tiff]

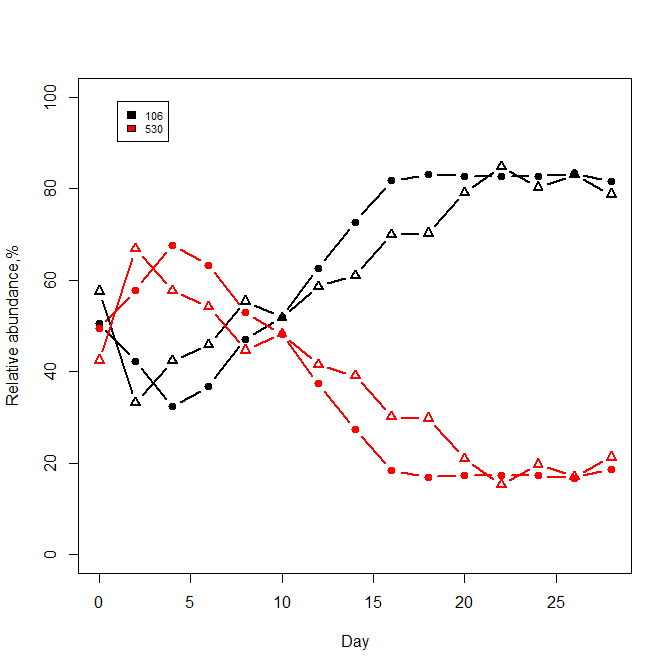

Supplement: Figure S9 — Cultures were sampled for relative abundance measurement every two days but no fresh media was added to the cultures. The two independent replicates are represented by solid circles and open triangles. [file pone.0067210.s009.tiff]

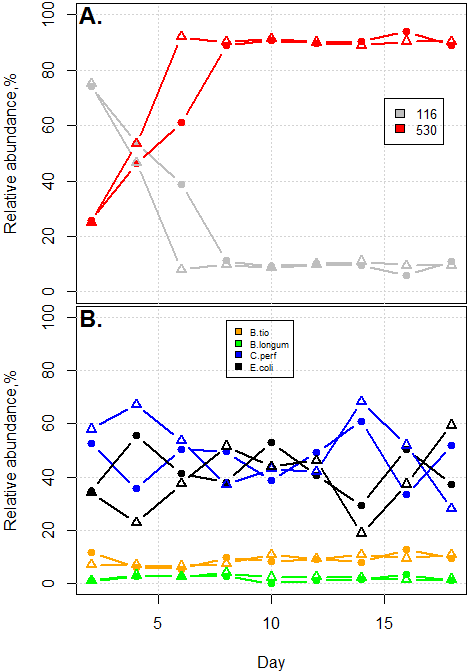

Supplement: Figure S10 — Competition between C. perfringens (C.perf), B . thetaiotaomicron (B.tio), B . longum and E. coli strains EDM116 and EDM530. Each experiment was performed in duplicate. (A) E. coli strain competition trajectories. (B) Relative species abundances at corresponding time-points. Batch culture competitions were performed in Oxoid anaerobe basal broth. The two independent replicates are represented by solid circles and open triangles. [file pone.0067210.s010.tiff]

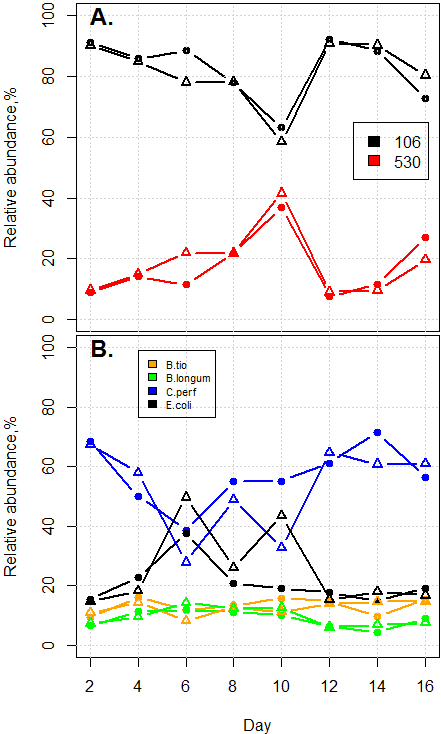

Supplement: Figure S11 — : Intra-specific competition is modulated by the resident background community. Percent relative abundances are plotted as a function of time for the competitions between C. perfringens (C.perf), B . thetaiotaomicron (B.tio), B . longum and E. coli strains EDM106 and EDM530. Experiments were carried out in duplicate. (A) Relative E. coli strain abundances. (B) Relative species abundances. After day ten, competitive strain trajectories change, coinciding with C. perfringens dominance. The two independent replicates are represented by solid circles and open triangles. [file pone.0067210.s011.tiff]

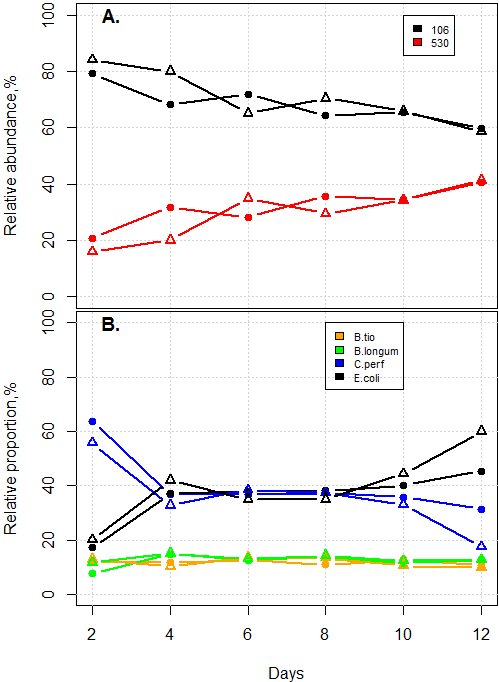

Supplement: Figure S12 — Percent relative abundances are plotted as a function of time for the competitions between C. perfringens (C.perf), B . thetaiotaomicron (B.tio), B . longum and E. coli strains EDM106 and EDM530. Experiments were carried out in duplicate. (A) Relative E. coli strain abundances. (B) Relative species abundances. After day ten, competitive strain trajectories do not change. This result occurs despite the higher aerobic growth rate of strain EDM106 relative to EDM530. The two independent replicates are represented by solid circles and open triangles. [file pone.0067210.s012.tiff]

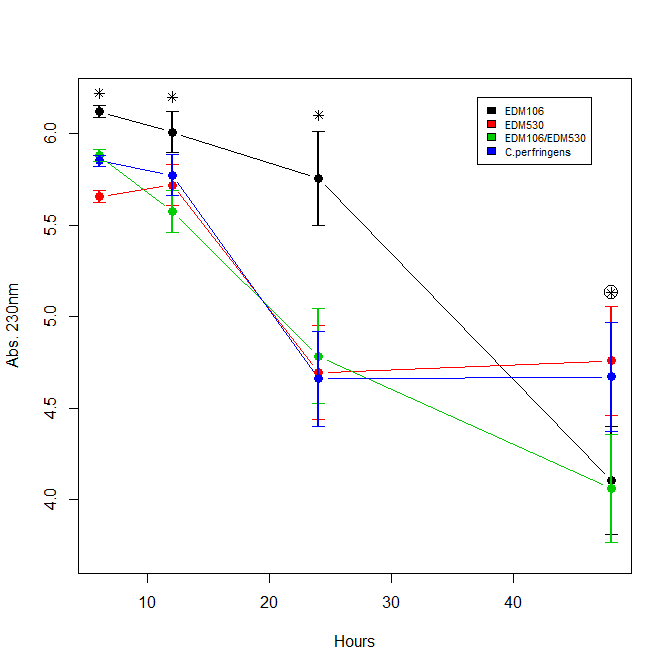

Supplement: Figure S13 — Overnight cultures were inoculated into fresh medium and samples were taken at 6, 12, 24 and 48 hours. Absorbance at 230nm was measured over time to determine peptone concentrations (see File S4). In the EDM106 culture peptone levels were consistently higher throughout the first 24 hours compared with cultures of EDM530, EDM530/EDM106 co-culture, and C. perfringens (asterisk indicates p<0.05). At 48hours, peptone levels were lower in the cultures containing EDM106 than cultures without EDM106 (circled asterisk indicates p<0.05). Glucose concentrations were also measured but were below the linear detection range of the assay for all strains by 6hours. [file pone.0067210.s013.tiff]

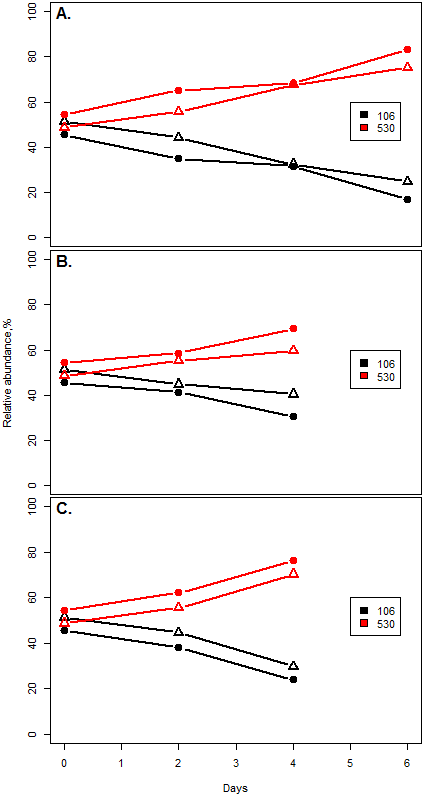

Supplement: Figure S14 — Investigation into potential factors released into the media was performed by growing C. perfringens to saturation (14 hours) in Oxoid anaerobic basal broth and then removing cells by filtration to create a spent rich medium. (A) 90/10, (B) 50/50, or (C) 10/90 (spent/fresh) medium proportions were then used for the E. coli strain competitions. The two independent replicates are represented by solid circles and open triangles. [file pone.0067210.s014.tiff]

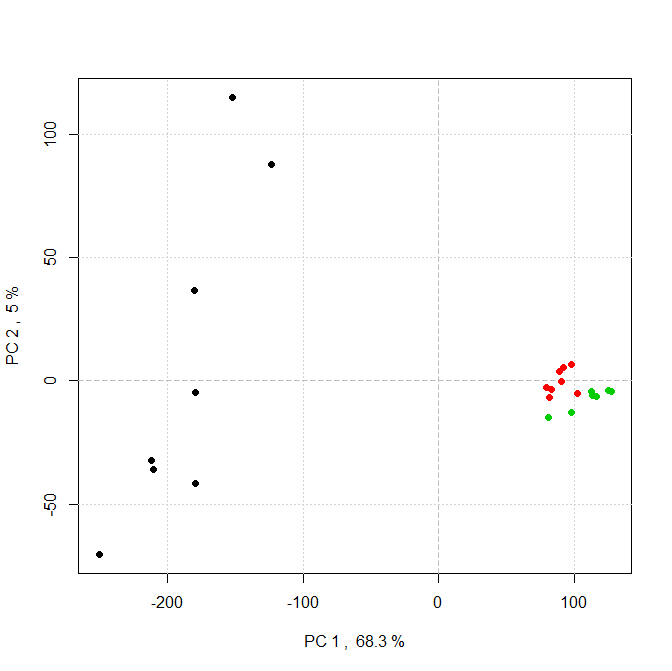

Supplement: Figure S15 — PCA of normalized HNMR spectra of competition time points in 90/10 (black dots), 50/50 (red dots), 10/90 (green dots) minimal salts (W)/ Oxoid anaerobic basal broth (rich medium). Clustering differentiates the spectra of the 10/90 and 50/50 (W)/rich medium competition supernatants compared with 90/10 medium. [file pone.0067210.s015.tiff]

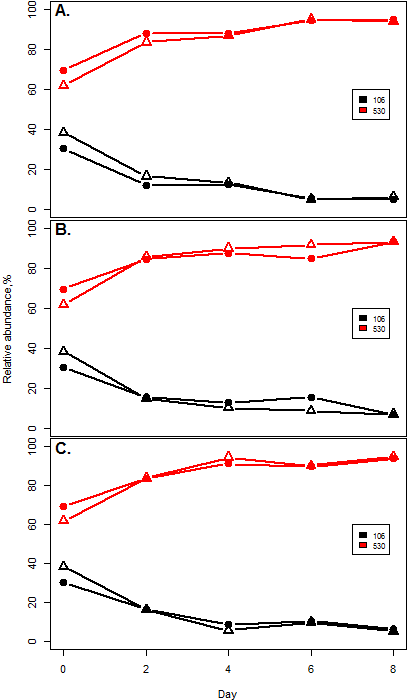

Supplement: Figure S16 — Minimal salts medium with different amounts of glucose as the sole carbon source was used for the competitions (Table S2). Revived frozen stocks from day 10 of strain EDM106 and strain EDM530 competition were used to start the competition. After two days equilibration in Oxoid anaerobic basal broth (rich medium), aliquots were transferred into either (A) 90/10, (B) 50/50 or (C) 10/90 glucose/minimal salts medium, relative to the amount of glucose in the rich medium (Table S2). The two independent replicates are represented by solid circles and open triangles. [file pone.0067210.s016.tiff]

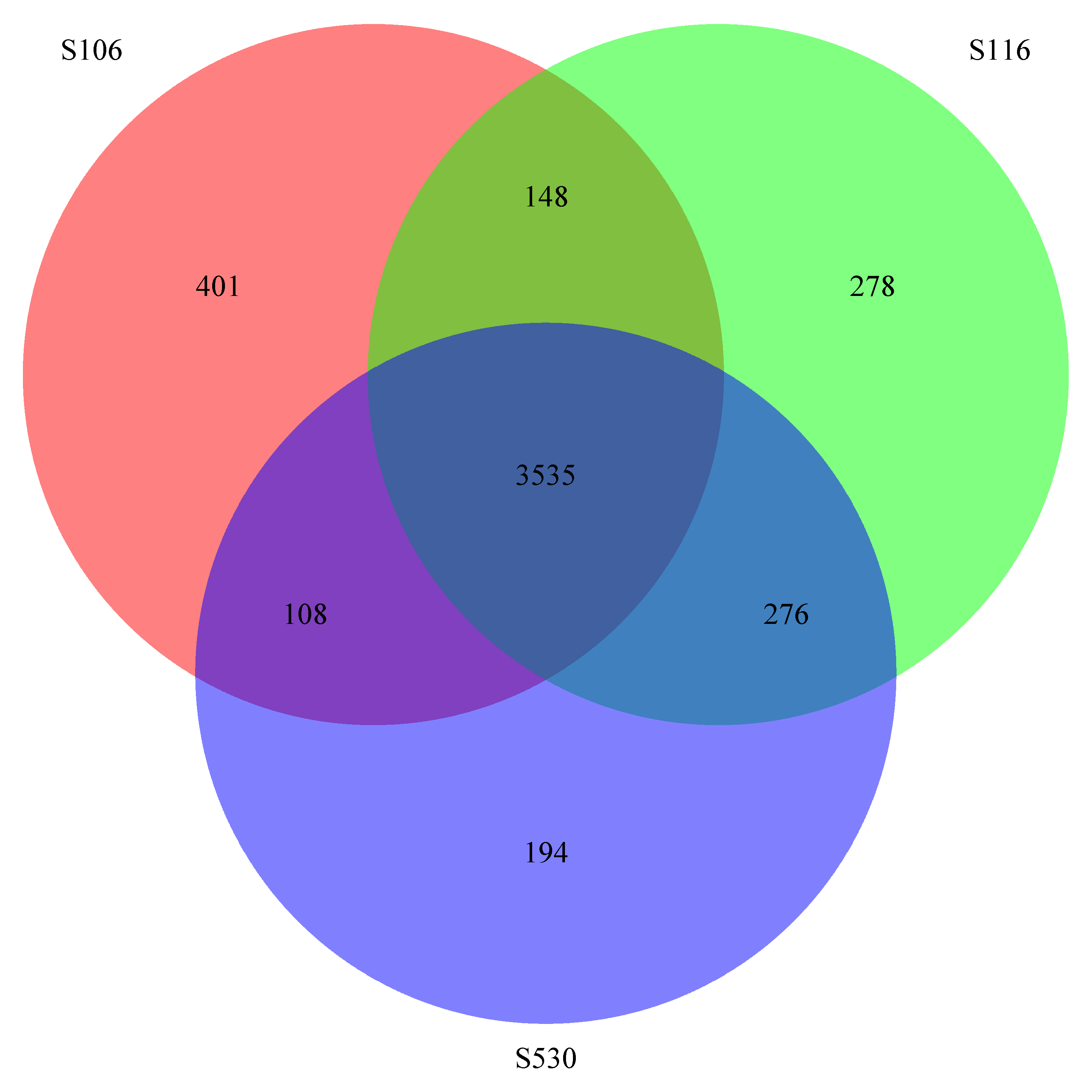

Supplement: Figure S17 — The three strains had totals of 4,192 genes (S106), 4,237 genes (S116) and 4113 genes (S530). The relative percents of unique genes found S106 with the largest (9.7%), 6.6% for S116, and S530 had the least (4.7%). Core genes represented 72% of the annotated total. See Supporting Data Files S1, S2 and S3 for listings of non-core gene annotations. [file pone.0067210.s017.tiff]

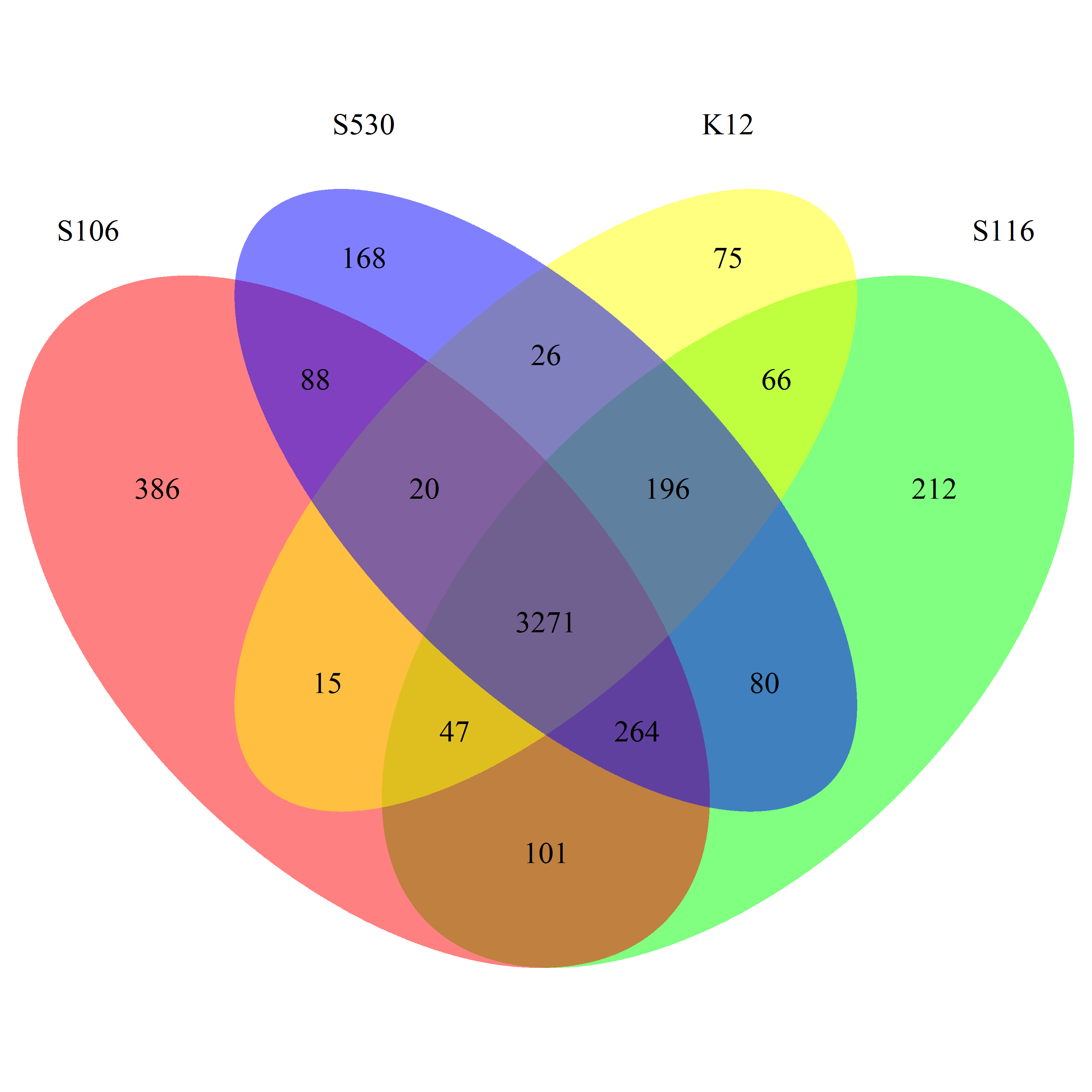

Supplement: Figure S18 — The three strains had totals of 4,192 genes (S106), 4,237 genes (S116) and 4113 genes (S530). The relative percents of unique genes found S106 with the largest (9.7%), 6.6% for S116, and S530 had the least (4.7%). Core genes represented 72% of the annotated total. [file pone.0067210.s018.tiff]
