# Supplementary material for: Context-Dependent Competition in a Model Gut Bacterial Community
Source: PLoS One. 2013 Jun 14;8(6):e67210. doi: 10.1371/journal.pone.0067210 (PMC3683063; doi:10.1371/journal.pone.0067210)
Supplement: File S4 [file pone.0067210.s025.docx]

**Supplementary Methods:**

*Batch culture competitions*

All competition experiments were carried out in 100ml flasks containing 90ml of medium and a water trap to allow gas to escape but kept the flasks anaerobic after the oxygen in the head space was consumed. The only exception to this protocol was the experiment shown in Supplementary Figure 11, where we attempted to give *E.coli* more of a competitive advantage relative to the background flora. In this experiment the water traps were emptied in order to allow small amounts of oxygen to enter the flasks. Flasks were incubated at 125rpm and 37ºC. Oxoid anaerobic basal broth was either prepared fresh or boiled in a water bath for at least ten minutes to de-gas the media. A typical experiment (2 replicates) used 90 ml of media per replicate for each time point. Mostly, 500ml batches of media were prepared but sometimes as much as 1 liter was made. This required many separate batches of media to be used throughout most of the individual experiments and also across the entire study. At the beginning of each of the competition experiments, the same inoculum was diluted into each of the replicate flasks. Thereafter, 100µl of the cultures were transferred to flasks containing fresh medium. This procedure established two independent but parallel series of competition time points. The competitions of strain EDM106 and strain EDM530 presented in figures 2A, 2B, 3, 4, S9, S14 and S16, are continued from revived frozen stocks from day 10 of the two day strain EDM106 and strain EDM530 competition presented in figure S4C. Aliquots were revived and continued for two days in complete Oxoid anaerobic basal broth before individual competition treatments were performed. Long term stationary phase cultures used the same conditions as described above, except that no transfers to fresh medium was carried out and medium was not replenished after inoculation. The peptone media experiments were conducted using different concentrations of peptone and the individual experiments were performed using two different brands of peptone media. Experiments shown in Figure 4A-C were conducted using Bacto™ Peptone manufactured by BD Biosciences. Experiments shown in Figure 4D-F were conducted using Fluka peptone from Sigma-Aldrich. Both types of peptone are enzymatic digests of animal protein.

*Spiking E.coli co-cultures with C.perfringens or B.thetaiotaomicron*

These experiments were started from revived frozen stocks from day 10 of the two day strain EDM106 and strain EDM530 competition presented in figure S4C. Aliquots were revived and continued for two days in complete Oxoid anaerobic basal broth before individual competition treatments were performed. On day 12 1ml of overnight culture from either *C.perfringens* or *B.thetaiotaomicron* was transferred to flasks with fresh medium along with 100µl of *E.coli* co-culture, thus providing a 10:1 by volume starting ratio between the respective species. Samples were then taken out as usual starting on day 14.

*Measurement of medium carrying capacity*

Strains were grown to saturation (16hour culture) in Oxoid anaerobic basal broth. 90ml of culture was separated into three 50ml conical vials with perforated tops of 30ml each and freeze dried. After freeze drying, all tubes were allowed to equilibrate to atmospheric moisture levels for 1week before weighing. The t.test that is presented is of strain EDM106 against the EDM106/EDM116 mixed culture.

*Mixed effects modeling*

The mixed effects model was fitted using the nlme package for R. Logit-transformed proportions were modeled as a function of the interaction of time and treatment (peptone concentration) assuming non-random intercepts and fitting a first-order continuous autoregressive process in the errors. The test was done for unequal slopes of strain proportions between the low (1.6 g/L) and high (14.4 g/L) peptone competitions for the first experiment (Figure 4A and C), and similarly between the low (3.2 g/L) and high (32 g/L) peptone competitions for the second experiment (Figure 4D and F).

*Measurement of Peptone and Glucose usage*

Overnight cultures of the individual strains were prepared in fresh Oxoid anaerobe basal broth and incubated at 37ºC, 125rpm. A single batch of Oxoid anaerobe basal broth was prepared and 100ml aliquots were portioned into 12 flasks (an increased volume compared with the other competitions to reduce head space and allow for repeated sampling from the flasks). 100µl (50µl of both EDM106 and EDM530 for the co-culture) from the overnight cultures were inoculated into the flasks in triplicate. 2ml samples were retrieved from the flasks at the indicated time points and spin at 3600rpm, 4ºC for 15minutes. The supernatant was removed and frozen at -80 ºC for later analysis. After thawing, samples were spun again at 7600rpm, 4ºC for 10minutes and the supernatant was diluted 1:10 in ddH2O prior to 230nm absorbance measurement using a NanoDrop, (Wilmington, DE, USA) ND-1000 spectrophotometer. Glucose was measured using the Glucose (GO) Assay Kit, SIGMA, Saint Louis Missouri, USA. Lower detection limit of the kit is 20µg glucose/ml. All samples measured below this level by 6 hours.

*Characterization of shared and unique gene content*

Due to the large number of contigs, determination of gene presence included additional processing steps *(in press).* Briefly, in order to recover genes split into separate contigs or genes that did not receive an annotation from RAST due to sequencing errors, we carried out an additional blast search using annotations of sixteen *E.coli* genomes. If any annotated gene was found in a subset of the genome collection, this was re-BLASTed against the raw assemblies of the strains not included in that subset. Re-assignment of the recovered sequence to the annotation required 90% identity and an e-value of <1e^-25^. Genes with redundant functional RAST assignments were collapsed into one single assignment prior to further analysis.
