# Supplementary material for: Context-Dependent Competition in a Model Gut Bacterial Community
Source: PLoS One. 2013 Jun 14;8(6):e67210. doi: 10.1371/journal.pone.0067210 (PMC3683063; doi:10.1371/journal.pone.0067210)
Supplement: Supplementary file 6 [file pone.0067210.s019.docx]

Isolate number	Age at sampling	Aero	s.e	Anaero.	s.e.	Colonization Catagory
EDM24	7 days	39.91	0.1369	48.71	0.6465	Early*
EDM123	4 months	40.75	0.5328	55.75	1.116	Late
EDM530	2 years	41.56	0.7187	49.03	0.2424	Late
EDM124	8-10 days	38.12	1.082	49.98	1.79	Early
EDM70	10 days	39	0.3685	53.86	0.1729	Early*
EDM71	10 days	39.57	0.4597	54.54	0.4518	Early*
EDM49	4 days	40.62	0.3767	55.21	0.2947	Early
EDM1.2	10 days	40.13	0.4572	52.56	0.3634	Early
EDM111	1 year	46.85	0.1906	63.99	0.5959	Late
EDM130	10 days	39.72	0.4042	53.88	0.3736	Early
EDM3	1 year	39.1	0.2759	50.32	0.3407	Late
EDM51	7 days	41.13	0.1506	50.92	0.8549	Early
EDM10	1 year	42.45	0.6447	52.04	0.7133	Late
EDM6	11 days	38.18	0.2411	51.57	0.6825	Early
EDM21	2 years	41.11	0.2318	56.91	0.1064	Late*
EDM5	1 year	42.74	0.5692	52.34	0.4431	Late
EDM101	11 days	41.97	0.6982	57.15	0.1964	Early
EDM69	2 years	42.83	0.1181	57.58	1.319	Late*
EDM11	1 year	38.26	0.5393	45.57	0.4071	Late
EDM131	1 year	38.61	0.1038	51.71	1.342	Late
EDM16	7 days	39.17	0.1958	48.26	0.128	Early*
EDM110	1 year	40.66	0.5612	51	0.4244	Late
EDM116	1year	40.98	0.4935	50.97	0.3206	Late*
EDM106	4 days	38.12	0.3495	56.5	0.3043	Early
EDM103	4 days	40.91	0.1273	54.05	0.4431	Early
EDM108	1 year	41.28	0.4512	53.51	0.8802	Late*
EDM118	4 days	39.93	0.3581	50.94	0.6398	Early
