# Supplementary material for: Context-Dependent Competition in a Model Gut Bacterial Community
Source: PLoS One. 2013 Jun 14;8(6):e67210. doi: 10.1371/journal.pone.0067210 (PMC3683063; doi:10.1371/journal.pone.0067210)
Supplement: Supplementary file 7 [file pone.0067210.s020.docx]

		Contig		
	Contig nr.	Length	depth	Best BLAST score
Strain 106	542	1527	433.9	E.coli plasmid pKL1, pO26-S1, pMG828-1, pSERB2 RepA and DfrA genes
	584	7968	162.4	E.coli SMS-3-5 plasmid pSMS35_8, pKY1 (from S.sonnei) cea, immunity protein (imm), kil genes, pColE1-EC39
	585	4078	137.3	E.coli SE11 pSE11-6 DNA, SMS-3-5 pSMS35_4, pIGWZ12, pMG828-2, O111:H- str. 11128 pO111_4
	563	1672	61.1	16s rRNA gene
	380	3245	58.9	23S rRNA gene
Strain 116	428	738	212.8	InsAB' transposase, IS1 protein InsA, IS1 protein InsB, plasmid associated
	863	4233	84.8	Associated with plasmids in Klebsiella, Salmonella, E.coli and Shigella, poor query coverage
	862	5512	80.7	E.coli pECO29, Salmonella enteritidis serovar Enteritidis pC
	444	2410	73.1	E.coli UM146 plasmid pUM146, E.coli strain CFT073 pathogenicity island II
	412	2454	59.1	E.coli O26:H11 str. 11368 DNA hypothetical protein, O111:H- DNA, genomic island GEI4.52, E. coli CFT073 hypothetical protei
	795	1472	58.3	16S rRNA gene
	464	3238	52.5	23S rRNA gene, 5S rRNA gene
Strain 530	75	1286	145.1	transposase IS116/IS110/IS902 family protein
	93	3103	126.2	23S rRNA gene
	163	1683	117	16S rRNA gene
	42	767	68.9	IS1 insertion element
	81	1010	67.9	transposase IS3/IS911 family protein
	74	472	65.2	rshA, possible capsular biosynthesis
