# Supplementary material for: Context-Dependent Competition in a Model Gut Bacterial Community
Source: PLoS One. 2013 Jun 14;8(6):e67210. doi: 10.1371/journal.pone.0067210 (PMC3683063; doi:10.1371/journal.pone.0067210)
Supplement: Supplementary file 8 [file pone.0067210.s021.docx]

Media Preparations

Oxiod anaerobic

basal broth (rich media)
